# Subthreshold Micropulse Laser Modulates Retinal Neuroinflammatory Biomarkers in Diabetic Macular Edema

**DOI:** 10.3390/jcm10143134

**Published:** 2021-07-15

**Authors:** Luisa Frizziero, Andrea Calciati, Giulia Midena, Tommaso Torresin, Raffaele Parrozzani, Elisabetta Pilotto, Edoardo Midena

**Affiliations:** 1 Department of Neuroscience—Ophthalmology, University of Padova, 35128 Padova, Italy; lfrizziero@gmail.com (L.F.); andrear.cal@gmail.com (A.C.); tommaso.torresin@gmail.com (T.T.); raffaele.parrozani@unipd.it (R.P.); elisabetta.pilotto@unipd.it (E.P.); 2 IRCCS, Fondazione Bietti, 00198 Rome, Italy; giulia.midena@gmail.com

**Keywords:** subthreshold micropulse laser, diabetic inflammation, diabetic retinopathy, diabetic macular edema

## Abstract

Subthreshold micropulse laser treatment has become a recognized option in the therapeutic approach to diabetic macular edema. However, some yet undefined elements pertaining to its mechanism of action and most effective treatment method still limit its clinical diffusion. We reviewed the current literature on subthreshold micropulse laser treatment, particularly focusing on its effects on the modulation of retinal neuroinflammation. Subthreshold micropulse laser treatment seems to determine a long-term normalization of specific retinal neuroinflammatory metabolic pathways, contributing to the restoration of retinal homeostasis and the curtailing of local inflammatory processes. Optimized and standardized parameters ensure effective and safe treatment.

## 1. Introduction

Diabetic macular edema (DME) is one of the leading causes of legal blindness in the worldwide working-age population. The pathophysiology of DME is complex and multifactorial and is not yet completely understood. Thus, DME represents one of the major therapeutic challenges for ophthalmologists [1,2].

Retinal microangiopathy, inflammation and neurodegeneration have been recognized as the major pathophysiological mechanisms leading to DME [2]. A growing body of evidence confirms DME as a manifestation of a chronic low-level inflammation that develops locally, at the retinal level, with the activation of retinal glial cells, the release of different cytokines and chemokines, neuronal cell death, and macular morphological and functional alteration [2]. 

Nowadays, the main treatment for DME consists of anti-vascular endothelial growth factor (VEGF) and steroid intravitreal injections. However, intravitreal treatment requires frequent injections administered in an appropriate setting, causing a significant burden for the patients and the healthcare system, and carries some risks related to the invasiveness of the procedure. Moreover, a significant percentage of DME patients show an absent or limited response to intravitreal anti-VEGF alone, highlighting the need for more tailored treatment strategies [3]. 

Standard (modified ETRDS) retinal laser photocoagulation has been used for a long time as the first-line treatment for DME, but it was later superseded by intravitreal treatment because of its greater visual improvement and fluid resorption, without laser local adverse events such as chorioretinal scarring and progressive retinal atrophy [4,5]. Subthreshold micropulse laser (SMPL) represents a tissue-sparing laser treatment modality, aiming at specific retinal targets, that avoids the drawbacks of photocoagulation [1]. Recently, a growing number of studies have confirmed the efficacy of SMPL in DME treatment. However, its main mechanism of action is still controversial. The aim of the present work is to summarize the current role of SMPL in DME treatment on the basis of the current literature.

## 2. Materials and Methods

To identify potentially relevant articles in the medical literature, we searched MEDLINE for English language articles published from January 1980 to December 2020. MEDLINE was queried using the following search terms (used both individually and in combination for advanced research): subthreshold laser, micropulse laser, diabetic macular edema, diabetic retinopathy. Additional articles were identified by reviewing the references of examined publications. To identify potentially relevant articles to include in this review, two investigators reviewed each paper. Case series were preferred to single-case reports. Articles included in the reference list were fully examined by the authors.

## 3. Results

### 3.1. Mechanism of Action

Subthreshold micropulse laser is designed to deliver energy within a pre-defined fraction of the total exposure time, with a fixed sequence of ON and OFF intervals. The ratio between the ON time and the exposure time (ON + OFF) is defined as a duty cycle, representing the effective delivery time of the laser. The individual spots are, by definition, invisible, using not only ophthalmoscopy but any currently available multimodal imaging retinal diagnostic techniques (e.g., spectral domain-optical coherence tomography (SD-OCT), OCT angiography, fundus autofluorescence, and retromode imaging), and never creates a local reduction of retinal sensitivity when examined with microperimetry [6,7]. Several functional tests (e.g., pattern electroretinography, automated microperimetry, a central vision analyzer) proved the ability of SMPL not only to maintain retinal function but also to improve it, not only in diabetic macular edema but in other retinal diseases such as age-related macular degeneration and inherited photoreceptor degeneration [8,9].

The currently commercially available devices for SMPL treatment include the infrared (810-nm) diode laser and the yellow laser that emits at a wavelength of 577 nm and has become the most widespread laser system [7,10].

The term subthreshold laser treatment defines a treatment modality, with still partly unknown chemico-physical effects, that does not produce any visible intra-retinal (as previously defined) change or damage during or after laser application [11]. Traditional suprathreshold laser treatment causes a visible (at ophthalmoscopy) burning effect, with subsequent tissue necrosis and scarring in the burned area. Moreover, it damages the surrounding tissues in a time-delayed manner, causing a lethal lesion that is below the threshold for visible necrosis and is therefore initially invisible, but manifests later as an atrophic scar. Finally, areas that are even further away from the suprathreshold delivery spot are subjected to negative effects—below any visible level—but sufficient to produce sublethal cellular modifications, as shown by retinal sensitivity alterations observed using microperimetry [11].

The use of micropulse laser treatment was first described in 1997, reporting its clinical efficacy in the treatment of macular edema using an 810-nm diode laser [12]. Early reports attributed the efficacy of SMPL to changes induced in the retinal pigment epithelium (RPE). In fact, the application of a micropulse subthreshold pulse (duty cycle < 20%) seemed to limit the spreading of the laser effects into the adjacent neurosensory retina and choroid [7,13]. A repetitive series of very short energy transmission periods, in a single spot, instead of a continuous-wave laser pulse, is used to deliver a total amount of energy that is insufficient to cause tissue damage [10]. The released laser energy stimulates cells, without structural side effects [4]. This leads to the positive modulation of retinal biological processes, such as inflammation, with the subsequent restoration of the blood-retinal barrier, which is now well known to be regulated by the retinal glial cell population [1,14]. SMPL downregulates, as recently demonstrated in humans, a series of local factors (growth factors and inhibitors, permeability factors, etc.) that addresses the underlying pathologic imbalance secondary to retinal chronic hyperglycemia [1,10,14].

Studies performed in vitro and on animals showed a series of retinal glial cell-derived cytokines and extracellular matrix-mediated responses [15]. Experiments on rabbits and mice proved that heat shock protein (HSP) expression after subthreshold laser irradiation did not significantly differ from other more damaging energy settings [16]. HSPs are constituents of a complex cellular defense mechanism and are induced by a variety of stressful stimuli, such as hyperthermia, hypothermia, ischemia, hypoxia, the depletion of ATP, free radicals, desiccation, viral infection, steroid hormones and ethanol [17]. Therefore, their induction by subthreshold stimulus confirmed that destructive irradiation is not necessary to obtain a biological response and maximize the release of HSP as a possible anti-inflammatory mediator [15,16,18]. Moreover, the modification of gene expression mediated by the cellular healing response to laser stimulus suggests that a therapeutic cellular cascade is activated, not by laser-killed cells, but by the still-viable cells that are reached by the laser energy and are exposed to sublethal insult [18,19]. The majority of these studies have been performed on animal models and, thus, do not fully represent the human retinal response to SMPL [16,17,20]. Therefore, new approaches have been studied to obtain a more direct and comprehensive understanding of the changes induced by SMPL in DME eyes. 

Recently, proteomic studies on ocular fluids, such as vitreous and aqueous humors (AH), gained greater relevance in the study of the pathophysiology and the response to treatment of several ocular disorders [21,22]. AH sampling, which is much more accessible than vitreous humor, has shown highly promising results in the proteomic investigation, not only for anterior segment disorders but also for posterior pole diseases, since AH and vitreous protein concentrations are strongly correlated [22]. Therefore, AH proteomic analysis has become a reliable search procedure to detect specific biomarkers in an easily accessible ocular fluid [21,22].

Aqueous humor sampling performed on DME eyes treated with 577-nm yellow light SMPL (5% duty cycle) allowed researchers to quantify the concentration of retina- and RPE-related biomarkers before and after SMPL treatment. The AH concentration of some RPE markers remained unchanged after successful treatment [21]. More specifically, pigment epithelium-derived factor (PEDF), an anti-angiogenic and neuroprotective factor secreted by RPE toward the neuroretina, and erythropoietin (EPO) represent two specific biomarkers of RPE activity [23,24]. Intraocular concentration, including aqueous humor levels, of these factors has already been reported as significantly modified in DME eyes compared to controls (PEDF reduction and EPO increase) [21,23,25]. However, the AH concentration of both these molecules showed non-significant variations after SMPL treatment at the one-year follow-up [21]. Since a statistically significant change of their concentration compared to baseline was never detected, their biology seems to be not significantly influenced by SMPL, at least as detectable in AH [21]. Conversely, another study reported a reduction of VEGF AH concentration after SMPL at 3 and 12 months versus the baseline, showing no statistically significant difference of mean VEGF concentration between diabetic and healthy subjects at the last follow-up [1]. This data suggests a progressive and long-term normalization of VEGF levels, secondary to SMPL treatment. Moreover, the same study found a modification of glial fibrillary acidic protein (GFAP) and inwardly rectifying potassium (Kir) 4.1 concentrations in patients with DME who were treated by SMPL [1]. Previous studies have already shown an increase in GFAP, aquaporin (AQP) 4, and specific cytokines in the AH of diabetic human eyes, even without signs of DR or at early stages of DR, as a sign of retinal glial cell activation [26]. The glial cells family includes Müller cells and astrocytes (macroglia) and microglial cells. They have a structural role, but also actively maintain the homeostasis of the retinal environment. Müller cells, connecting retinal neurons and vessels, are specifically involved in the retinal water control and inflammatory response, especially when activated by chronic hyperglycemia. Müller cells perform an active crosstalk with microglial cells to maintain physiological intraretinal homeostasis [27].

Microglial cells are considered the local immune cells of the retina, similar to the central nervous system microglial cells. They are activated by stress conditions, such as chronic hyperglycemia, and are able to change their morphology and function [28]. In the healthy retina, microglial cells are predominantly located in the inner retinal layers, in a ramified resting status. When activated, for example in diabetes, microglial cells turn into an ameboid form, gain motility, migrate from the inner to the outer retina, and release pro-inflammatory and vasoactive substances, such as VEGF, contributing to the local inflammatory response, followed by increased vascular permeability [26,28]. It has been proposed that microglia cells may be visualized in the retina using SD-OCT as discrete hyperreflective foci (HRF) [29]. Recently, SMPL was shown to reduce HRF even at a long-term follow-up (1 year), together with a reduction in the area of cysts, the disorganization of inner retinal layers and the number of microaneurysms [30].

GFAP is an intermediate filament protein that is expressed in large amounts in activated retinal Müller cells [27]. Its concentration has been shown to decrease at 12 months from the first SMPL application in DME eyes, due to the normalization of Müller cells. A similar reduction was found for Kir 4.1 [1]. This transmembrane protein is expressed in Müller cells, regulating K^+^ conductance and strongly influencing simultaneous water transport across the cellular membrane, coupled with AQP4. It has been found that Kir 4.1 is mislocated in Müller cells and functionally inactivated in different retinal disorders, including DR. The reduction of K+ current causes the rapid osmotic swelling of Müller cells. A progressive reduction of Kir 4.1 in patients with DME treated with SMPL has been reported at 1-, 3-, and 12-month follow-ups [1]. The reduced expression of both GFAP and Kir 4.1 suggests that SMPL may contribute to reducing the concentration of inflammatory cascade proteins produced by retinal glial cells [1,14]. An increased concentration of specific inflammatory proteins was found in diabetic patients with DME, such as regulated upon the activation of normal T-cells that are expressed and secreted (RANTES), macrophage inflammatory proteins (MIP1α) and fas ligand (FasL). All these factors showed a significant reduction in concentration after SMPL treatment, which proved to be able to induce a downregulation of inflammatory retinal processes [14]. As already mentioned, (neuro)inflammation has been recently recognized as one of the most important drivers in the pathogenesis of DME [2]. Therefore, these results strongly suggest that SMPL de-activates retinal glial cells and reduces the production of cytokines and chemokines, including VEGF, also restoring the integrity of the retinal structural and vascular morphology [1,14,30].

### 3.2. SMPL Technique

A wide range of treatment parameters and regimens have been reported in studies using SMPL. However, optimized and standardized parameters and treatment guidelines are necessary for tissue injury minimization and vision restoration. The main principles of subthreshold laser treatment have been delineated by the International Retinal Laser Society (LIGHT): high-density treatments with fixed laser parameters maximize therapeutic clinical effects, minimizing variability and being less dependent on the operator’s skills, even when applied in a confluent manner. Moreover, longer wavelengths, longer pulse durations, and low-frequency pulse trains are recommended to broaden the therapeutic range and increase safety [31]. In fact, a low duty cycle ensures an adequate molecular relaxation time between micropulses, reducing the risk of any lethal retinal injury. The LIGHT guidelines reported that the number of spots depends on the spot size; however, a small spot size produces a targeted and precise treatment. [4,11,31,32]. Safety standards have been defined by the American National Standards Institute (ANSI) using the concept of maximum permissible exposure (MPE), usually taken as 10% of the power or energy density that has a 50% probability of causing damage. According to this concept, power levels between 750 and 950 mW for an 810-nm laser, and 200–300 mW for a 577-nm laser, have been proved to be safe and effective in several studies [4,33,34,35,36]. A titration strategy has also been proposed, consisting of reducing the laser power to 30–50% of the preliminarily detected threshold value [33]. However, this approach has not proved to be devoid of risk, leading to high variability of power values, depending, for example, on the area of the retina that has been tested to set the threshold value and on the operator’s skills. Therefore, current recommendations include the avoidance of titration [31]. Fixed safe parameters have been recently confirmed to provide non-inferior results compared to titration, with safer margins [32,33]. Finally, a confluent modality of pulse delivery on the whole macular area, with a high number of laser spots, is necessary to obtain a “mass effect” of cellular stimulation [34,37,38]. 

Studies comparing yellow 577-nm versus infrared 810-nm wavelength micropulse lasers (the previously most commonly used and studied laser for SMPL) showed both lasers to be safe in patients with mild center-involved DME, both preserving, and in some cases improving, retinal sensitivity of the treated macula, as measured through microperimetry [32,33]. Mathematical analysis of enzyme reaction kinetics suggested that 810-nm SMPL may have a wider therapeutic range/safety margin. Calculations showed that similar degrees of power increase do not cause any safety issue using an 810-nm laser but might result in retinal burns using a 577-nm laser, confirming the necessity of using published fixed laser parameter sets to avoid the likelihood of miscalculation and inadvertent retinal damage, especially when using 577 nm [39]. However, at present, the yellow laser system is more easily available, and more studies are confirming its efficacy and safety [35,40,41]. The treatment of the whole macular area in DME using the 577-nm yellow light laser has shown good results in terms of efficacy and safety, using a spot size of 100 μm, duration of 0.2 s, 5% duty cycle, power of 250 mW, applied in a high-density, fully confluent fashion [1,14,26,32,33]. These parameters were also used in the majority of studies analyzing the aqueous humor of DME eyes before and after SMPL treatment, thus confirming the biological efficacy of this “standard” and repeatable treatment [1,14,21]. 

### 3.3. Efficacy

The first report of DME patients treated with a micropulse laser, although without a subthreshold strategy, was published in 1997 and showed a reduction of macular edema in 87.5% of treated eyes [12]. Later, Laursen et al. compared the efficacy of standard SMPL and a conventional argon laser in a randomized trial and reported equal efficacy [42]. Then, Luttrull et al. reported the maintenance of visual acuity in 85% of eyes treated with SMPL, and the resolution of macular edema in 79% of patients [34]. Since then, different prospective randomized trials have reported an improvement in visual acuity and retinal thickness with SMPL, at least equal to conventional laser photocoagulation for DME [9,19,43]. Moreover, the reduction of retinal thickness on OCT following SMPL was shown to be maintained during follow-up [9,19]. Studies also proved the efficacy of SMPL in improving visual function without visual field loss, with an absence of scotomata and the preservation of contrast sensitivity [9,10,19,43]. The use of microperimetry in DME demonstrated stabilization or an increase in retinal sensitivity after SMPL compared to conventional laser [9]. 

One of the major theories on the mechanism of action of conventional photocoagulation in the treatment of macular edema is the oxygen theory. It suggests that photocoagulation reduces the metabolic demand of the treated tissue and increases the oxygen supply to the remaining tissue. However, molecular studies on various cell types reported the reduction and regulation of the inflammatory cellular response as one of the main physiological effects of low power laser irradiation [44,45]. The demonstration of comparable results between SMPL and conventional photocoagulation seems to confirm that the altered expression of various mediators including VEGF may be a major mechanism of effect in the reduction of DME [44,45]. Moreover, one of the drawbacks of a conventional pulse duration laser is the upregulation of inflammatory cytokines in the neurosensory retina, such as VEGF, interleukin 6 (IL-6), RANTES, and monocyte chemotactic protein 1 (MCP-1). Conversely, the short-pulse laser has been shown to induce fewer inflammatory cytokines in the neurosensory retina compared with a conventional pulse duration laser, in animal studies, and SMPL has proved to reduce their concentration in human aqueous humor [14,46].

After SMPL, the first structural and functional results appear at about three months. Luttrull et al. found that macular thickness did not change significantly in the first 2 months after treatment. At 3 months, however, a reduction in macular thickness was observed [47]. Moreover, an early increase in visual function from the third month after SMPL treatment in DME has been reported [48]. Lavinsky et al. showed a significant increase in best-corrected visual acuity from the third month after high-density SMPL [19]. An increase in central retinal sensitivity was also reported in the first months of follow-up after SMPL treatment [48]. Furthermore, in these studies, patients continued to improve at a long-term follow-up (1 year) [1,19,35,48]. These results may be due to progressive retinal metabolic restoration, whose long-lasting benefits might be mediated by changes in protein secretion (cytokines and growth factors), with a consequent downregulation of inflammatory processes and improvement of fluid homeostasis. 

In the majority of trials, the average central retinal thickness (CRT) prior to SMPL treatment was < 400 μm [19,35,48,49]. Mansouri et al. reported a significant difference in patients with a central foveal thickness (CFT) < 400 μm and CFT > 400 μm. The former experienced a mean reduction of 55 μm in CFT and an 0.2 log MAR gain in visual acuity at 12 months, without rescue anti-VEGF injections [6]. The latter showed no significant change in CFT or visual acuity by 6 months, despite retreatment, requiring anti-VEGF injections from 6 to 12 months of follow-up. The limited efficacy of SMPL in severe edema may be due to the altered distribution of SMPL energy throughout the retina, with the reduced possibility of restoring macro- and microglial functions [6,49].

Considering single retinal layer thickness, a previous study conducted on untreated center-involved diabetic macular edema with a central retinal thickness of 400 μm or less, and treated with SMPL, showed a significant decrease in inner nuclear layer (INL) thickness, and an increase in visual acuity and retinal sensitivity significantly and inversely correlated to CRT, INL, and outer nuclear layer (ONL) thickness [48]. Moreover, SMPL was shown to improve, firstly, visual function parameters, and then the structural ones [48]. The particularly significant effect of SMPL on INL thickness may indicate that SMPL has a major effect on the structure/function of INL resident cells, mostly (at least as far as volume is concerned) represented by Müller cells. As previously mentioned, these cells play an important role in the maintenance of normal retinal structure and function and are activated in response to specific types of retinal injury, such as chronic hyperglycemia [27]. Any “injury” stimulates the glial reaction, consisting of cellular hypertrophy, proliferation, and targeted cellular migration, leading to an increase in thickness of retinal layers, specifically INL, even in diabetic eyes without clinical signs of diabetic retinopathy [27,50]. The reduction of INL thickness supports the hypothesis that SMPL may induce the downregulation of Müller cell-related pathological processes, and the return of Müller cell bodies to normal size. The association of INL thickness reduction, detected at SD-OCT, combined with the reduction of concentration in AH samples of Müller cell- and inflammation-related factors, suggests that SMPL induces a functional and anatomical restoration of Müller cells, and thus affects the associated inflammatory intraretinal processes [1]. Moreover, regulation of the blood–retinal barrier is one of the functions of Müller cells. Therefore, the normalization of Müller cell activities is accompanied by mending of the blood–retinal barrier and edema reduction [27].

Recently, some reports suggest that SMPL may reduce the burden of intravitreal treatments and improve response, particularly in severe or refractory DME [3,40,41,51]. The association between anti-VEGF injections (Ranibizumab or Aflibercept) and SMPL (after the anti-VEGF loading phase) has been studied both retrospectively and prospectively. The main significant results were obtained long-term, with the reduction of the number of required injections at 12 months [3,40,41,51]. This may be due to the different main mechanisms of anti-VEGF and SMPL treatment: the first allows for a rapid beneficial effect, reducing vessel permeability and restoring macular integrity. Once the retinal thickness has been reduced, SMPL allows for a widespread cellular (mainly microglia and Müller cells) restoration, normalizing the complex mechanisms of regulation of inflammation, vessel permeability, immunomodulation, and tissue remodeling, as already suggested in 2015 by Luttrull et al., in age-related macular degeneration [38,52]. 

Therefore, SMPL effects may take a few months to become evident, but may allow for a consolidation of the morphological and functional results, and a reduction of the intravitreal injections burden.

### 3.4. Safety

Micropulse laser treatment using the abovementioned parameters has been shown to be a safe method, improving visual function and avoiding any visual field loss with the absence of scotomata, while preserving retinal sensitivity (as assessed by microperimetry) [34]. Moreover, studies using OCT, autofluorescence, OCT angiography, fluorescein and indocyanine angiography, and electroretinography have confirmed the absence of clinically detectable retinal and choroidal alterations after SMPL treatment [9,19,36,53].

Retinal changes have been reported in eyes treated with duty cycles ≥ 15% and laser power titration. In fact, this treatment modality may raise some concerns of overtreatment because of the difficulty in determining the correct retina position suitable for titration and the percentage of power to be reduced [32]. Moreover, the use of a finite-element model of 810-nm laser heating and damage to the human retina has shown that the use of a duty cycle ≥ 10% may also cause an increase in temperature, possibly exceeding the damage threshold, that may lead to an ophthalmoscopically or multimodal imaging–visible lesion. Therefore, the actual recommendation is for a low duty cycle, namely, 5% [54]. 

## 4. Conclusions

The subthreshold micropulse laser has become a recognized effective treatment for patients affected by mild DME. Growing evidence shows that a key element in this regard is the normalization of a series of cellular functions restoring the morphological and functional integrity of the retina. Among these, a primary role is held by the regulation of a series of growth factors and inhibitors and permeability agents, the improvement of macro- and microglial function, the increased expression of cytokine and interleukin markers of reparative acute inflammation, the decreased expression of markers of chronic inflammation, and tissue remodeling induced by stress conditions. The treatment involves different cellular elements, including glial cells, allowing for the long-lasting normalization of the intraretinal homeostasis. This evidence hints at the possibility of the wider use of SMPL, not only limited to mild DME but also as a key treatment option in the long-term management of earlier DME, or in combination in more severe and refractory DME cases.

## Data Availability

The data presented in this study are available in the article.
